# Evaluation of Cytokeratin-7 and Cytokeratin-19 Expression Relationship with Gleason Score in Prostatic Adenocarcinoma

**DOI:** 10.30699/IJP.2023.2002657.3116

**Published:** 2023-08-18

**Authors:** Masood Soltanipur, Mohammadreza Jalali Nadoushan, Hossein Yarmohammadi

**Affiliations:** 1 *Department of Pathology, Faculty of Medicine, Shahed University, Tehran, Iran*; 2 *Medical Students Research Committee, Shahed University, Tehran, Iran*

**Keywords:** Ck-7, Cytokeratin-19, Ck-19, Cytokeratin-7, Gleason Score, PAC, Prostatic adenocarcinoma

## Abstract

**Background & Objective:**

Prostatic adenocarcinoma (PAC) is one of the most common tumors worldwide. Immunohistochemical expression of cytokeratins has been evaluated in the diagnosis and prognosis of tumors. The aim of the present study is the evaluation of Cytokeratin-7 (Ck-7) and Cytokeratin-19 (Ck-19) expression and its relationship with Gleason score in patients with PAC.

**Methods:**

In this cross-sectional study, 78 samples from 78 patients with PAC referred to Mostafa Khomeini Hospital were gathered. Samples were immunohistochemically stained by Ck-7 and Ck-19 markers. The percentage of each marker in tumor cells was determined, and its relationship with Gleason scores and Gleason grade groups was analysed by SPSS version 24.

**Results:**

The expression of Ck-7 and Ck-19 were seen in 37.2% and 82.1% of samples, respectively. The mean of Ck-7 expression in tumor cells was 4.98%±7.19 (ranged 0 to 26%), while the mean of Ck-19 expression was 41.02%±23.36 (ranged 0 to 78%). There was no relationship between Ck-7 expression with Gleason scores and Gleason grade groups. However, Ck-19 expression was increased in higher Gleason scores and Gleason grade groups (*P*<0.001). No relationship was found between age and Ck-7 (*P*=0.309) and Ck-19 (*P*=0.375).

**Conclusion:**

The Ck-7 expression in PAC samples is weak and focal and had no relationship with the Gleason scores and Gleason grade groups. However, Ck-19 expression in PAC was high and was associated with tumor dedifferentiation of samples. There was no relationship between the expression of both markers with the patient's age.

## Introduction

Prostate cancer is the most common malignancy and the second leading cause of cancer-related death in men worldwide, accounting for approximately 29% of all cancers in the United States (1, 2). The incidence of this disease has a direct relationship with age, so prostate cancer diagnosis before the age of 50 is almost rare, and with aging, the incidence and mortality of the disease increase sharply. Prostate adenocarcinoma (PAC) is responsible for more than 90% of epithelial malignancies of this organ and originates from the glandular part of the prostate (3, 4). The Gleason score plays an essential role in predicting the prognosis of PAC and is one of the first and most successful variables in choosing the optimal treatment for patients with prostate cancer by minimizing treatment-related complications and maximizing treatment benefits (5, 6). The Gleason score is the strongest predictor of prostate cancer prognosis, which is defined as the sum of the scores of the first and second most common structures in the biopsy (7). This system is based on the glandular differentiation pattern of the tumor, and the characteristics of the cells do not play a role in it. A higher Gleason score has been proven as a marker of more aggressive biological behavior of the tumor (8).

An essential part of prostate cancer management is the early diagnosis of malignancy and its differentiation from other malignancies of the genitourinary system. The progression of normal prostate epithelial cells to the neoplastic state is a multi-step process characterized by continuous changes in cell phenotype. Biomarkers are needed to distinguish tumor tissue more effectively from normal prostate and identify therapeutic targets for the development of prevention and treatment (9, 10). In the last two decades, many tumor markers have been used as immunohistochemical aids for cancer diagnosis. Cytokeratins are one of the important and non-specific tumor markers that are expressed in various tumors including lung, breast, colon, prostate, and ovary. Cytokeratins are a group of water-soluble proteins that appear mostly in the epithelium and play a role in the structure of the nuclear matrix (11). The presence of keratin in malignant cells can be a useful marker to distinguish between epithelial tumors and tumors of endodermal, neuroectodermal, mesenchymal, or germ-cell origin. Diverse patterns of cytokeratins are associated with different pathways of differentiation and, as a result, enable the classification of epithelial cells into different subtypes (12, 13). The expression profile of cytokeratins is commonly used today to help diagnose the primary lesion as well as identify the primary location of metastatic carcinomas and determine the prognosis (14, 15). However, because still limited evidence has investigated the expression of Ck-7 and Ck-19 in prostate adenocarcinoma, this study aimed to evaluate the expression of these immunohistochemical markers in PAC samples and its relationship with the Gleason score of the tumor.

## Material and Methods

**Patients and Sample Collection**

To carry out this study, searching in the database of the Pathology Department of Mostafa Khomeini Hospital, Tehran, all the samples with the final diagnosis of PAC from radical prostatectomy and transurethral resection of the prostate during the years 2017 to 2022 were obtained. Clinical information including the age of the patients, was extracted from their files in the pathology department. Then, the paraffin blocks fixed in formalin and slides stained with the H&E method were reviewed by a pathologist and in addition to confirming the diagnosis and determining the primary, secondary, and total tumor Gleason scores and Gleason grade groups, the blocks with minimal necrosis and bleeding with enough tumor cells were selected for immunohistochemical staining. Samples that did not have enough tissue for staining or whose files were incomplete were excluded from the study. Finally, immunohistochemical staining for Ck-7 and Ck-19 was performed on the samples according to the instructions as follows.

**Immunohistochemical Studies**

First, two 4-micron slices were prepared from each paraffin block using a microtome. Then, for deparaffinization, the samples were placed in an oven at 60°C for 45 minutes. Then, the rehydration step was done by placing the samples in three containers of xylenol each for 10 minutes and then three containers of alcohol with concentrations of 100, 96, and 70%, respectively, and in each container for 5 minutes to prepare the tissue for staining. Then, to retrieve the antigen, the samples were placed in 1 M citrate buffer with pH=6 in an autoclave with a pressure of 15 atmospheres and a temperature of 126 degrees Celsius for 10 minutes. After washing the slides with TBS buffer with pH=7.2-7.6, one drop of peroxidase block solution was poured on the slides, and after 5 minutes, the slides were washed twice with TBS buffer and each time for 5 minutes. After this time, the surface of the slides was covered with protein blocking solution for 5 minutes, and after this period, the surface of the slides was washed twice with TBS buffer for 5 minutes each time. These actions were performed to remove excess antibodies. In the next step, Ck-7 and Ck-19 monoclonal antibodies (Biogenex, USA) were placed on the slides for one hour. After this time, the slides were washed twice with TBS buffer for 5 minutes each time. In the next step, to stain the slides, diaminobenzene chromogen was added to the resulting slides for 5 minutes, and then the slides were placed in hematoxylin for 30 seconds. To remove the excess colors, the slides were placed in the Xylenol solution for 3 seconds. Finally, the slides were mounted after drying and prepared for observation. Finally, slides stained with Ck-7 and Ck-19 antibodies were observed by a pathologist using a light microscope at 40X magnification. Membranous and cytoplasmic staining with each of these antibodies was considered a positive result, and the percentage of positive tumor cells was determined by the pathologist.

**Statistical Analysis**

The collected information was entered into SPSS version 24 statistical software (IBM SPSS Statistics, New York, United States) and subjected to statistical analysis. Qualitative and quantitative descriptive data were expressed as frequency percentages and mean ± standard deviation, respectively. One-way ANOVA and Kruskal-Wallis statistical tests were used to analyze the data and check the statistical relationship between the expression percentage of the examined markers and the Gleason scores and Gleason grade groups of the tumor. The *P*<0.05 was considered statistically significant in this study.

## Results

A total of 78 PAC samples from 78 patients were analyzed in this study. Table 1 shows the frequency of each of the primary, secondary, and total Gleason scores and Gleason grade groups in the studied samples, respectively. The average age of the patients investigated in this study was 70.8 ± 8.78 years, with a median of 72 years and a range of 53 to 89 years.

Based on the results of Ck-7 immunohist-ochemical staining in PAC samples, the expression was not observed in 49 samples (62.8%). The average expression of this marker in total tumor cells was 4.98% ± 7.19 and in the range of 0 to 26%. The expression status of the Ck-7 marker according to primary, secondary, and total tumor Gleason scores and Gleason grade groups is shown in Table 1.

The expression of Ck-19 was not observed in 14 samples (17.9%). The average expression of Ck-19 in all tumor cells in the examined samples was 41.02 ± 23.36% and in the range of 0 to 78%. The average expression percentage of Ck-19 according to primary, secondary, and total tumor Gleason scores and Gleason grade groups is shown in Table 1.

There was no statistically significant relationship between the expression of Ck-7 and the primary, secondary, and total tumor Gleason scores and Gleason grade groups of PAC. However, the percentage of Ck-19 expression increased significantly with the increase of primary, secondary, and total tumor Gleason scores and Gleason grade groups, so all negative tumors in terms of Ck-19 expression had total Gleason score of 4 to 6 and 1 Gleason grade group. 

The relationship between the expression of Ck-7 and Ck-19 markers in total tumor cells with age is shown in Table 2. Based on this, it was found that there was no statistically significant relationship between the expression percentage of both markers with the patient's age.

**Table 1:** The mean of Ck-7 and Ck-19 expression according to primary, secondary, and total Gleason scores and Gleason grade groups

Gleason (N, %)		Ck-7 expression (%)	Ck-19 expression (%)
Primary scores	2 (19, 24.3%)	5.63 ± 4.76	20.78 ± 17.18
	3 (31, 39.7%)	4.93 ± 6.13	30.58 ± 18.76
	4 (19, 24.3%)	5.31 ± 7.8	63.21 ± 10.06
	5 (9, 11.5%)	2.44 ± 6.33	74.88 ± 7.83
	P-value	0.283	<0.001
	Total	4.91 ± 7.35	41.25 ± 25.23
	Total range	0 - 27	0 - 85
Secondary scores	2 (16, 20.5%)	4.37 ± 3.71	22.12 ± 19.36
	3 (33, 42.3%)	6.09 ± 6.7	35.21 ± 23.02
	4 (23, 29.5%)	4.69 ± 7.38	55.3 ± 8.58
	5 (6, 7.7%)	3.83 ± 8.39	61.5 ± 13.61
	P-value	0.672	<0.001
	Total	5.15 ± 7.45	40.47 ± 22.55
	Total range	0 - 26	0 - 79
Total scores	4 (4, 5.1%)	5.5 ± 3.87	26.25 ± 17.51
	5 (21, 26.9%)	7.33 ± 6.35	17.33 ± 19.64
	6 (13, 16.7%)	4.15 ± 5.81	36.61 ± 18.75
	7 (20, 25.6%)	4.15 ± 6.8	51.35 ± 12.79
	8 (14, 17.9%)	4 ± 6.41	59.21 ± 12.53
	9 (6, 7.7%)	3.33 ± 7.16	66.5 ± 9.37
	P-value	0.286	<0.001
	Total	4.98 ± 7.19	41.02 ± 23.36
	Total range	0 - 26	0 - 78
grade groups	1(38, 48.7%)	6.05 ± 6.34	24.86 ± 20.69
	2(11, 14.1%)	2.45 ± 5.50	43.90 ± 9.03
	3(9, 11.5%)	6.22 ± 9.90	60.44 ± 10.84
	4(14, 17.9%)	4.00 ± 8.41	59.21 ± 12.53
	5(6, 7.7%)	3.33 ± 8.16	66.50 ± 9.37
	P-value	0.321	<0.001
	Total	4.98 ± 7.19	41.02 ± 23.36
	Total range	0 - 26	0 - 78

**N:** Number, **%:** Percent, **Ck-7:** Cytokeratin-7, **Ck-19:** Cytokeratin 19

**Table 2:** The mean of Ck-7 and Ck-19 expression according to age groups

Age groups (year)	Ck-7 expression (%)	Ck-19 expression (%)
50 - 60	4.53 ± 5.25	37.92 ± 25.92
61 - 70	3.72 ± 5.01	44.27 ± 20.18
71 - 80	4.7 ± 6.05	43.77 ± 23.73
81 - 90	8.5 ± 7.35	31.33 ± 27.25
P-value	0.309	0.375

**Ck-7:** Cytokeratin-7, **Ck-19:** Cytokeratin-19, **%: **Percentage

## Discussion

In the present study, the immunohistochemical expression of Ck-7 and Ck-19 markers in tissue samples of PAC with different Gleason scores and Gleason grade groups was investigated. According to the findings of the present study, the expression of Ck-7 was not observed in 62.8% of cases, while in 37.2%, a small expression of this marker was evident. Also, the maximum expression of this marker was observed in 26% of tumor cells.

Other studies conducted in the field of the expression of Ck-7 in PAC have often examined the simultaneous expression of this marker with cytokeratin-20 (Ck-20) or other markers to differentiate PAC from bladder urothelial carcinoma. In the study conducted by Gheitasi* et al.*, the expression of Ck-7 was present only in 27.8% of cases (16). Also, in the study of Mhawech* et al.*, which was conducted in the United States and Switzerland, the positive expression of Ck-7 was observed in 27.5% of PAC samples with a Gleason score of 8 or higher, and in 75% of cases, the expression of Ck-7 and Ck-20 were simultaneously negative (17). In a study published by Adisa* et al.* in Nigeria, the expression of Ck-7 was observed in 80% and 13.3% of benign prostate hyperplasia and PAC samples, respectively (18). In the study conducted by Goldstein* et al.* in the United States, Ck-7 expression was present in 49.8% of PAC cases with a Gleason score of 6 to 10, but in most samples, it was observed in less than 25% of tumor cells (19). In the study of Genega* et al.*, the expression of Ck-7 was observed in only 19% of PAC samples (20). In two other studies conducted in the United States, which examined the simultaneous expression of Ck-7 and Ck-20 in PAC patients, the absence of simultaneous expression of these two markers was observed in 81% and 86% of the samples, respectively. Additionally, only 10% of samples showed positive expression of Ck-7 (21, 22). Interestingly, the results of the study of Dariane* et al.* showed that Ck-7 is expressed in normal prostate tissue and benign glands around the tumor, mainly in the cells of the basal and suprabasal layers, but it is not present in the luminal cells of the tumor (23). Therefore, the expression of Ck-7 decreases during the development of PAC. Ck-7 expression is usually negative or shows weak and focal expression in PAC samples, which seems to be an aberrant expression.

In our study, although the expression of Ck-7 decreased slightly with increasing Gleason score and Gleason grade group of the tumor, the findings did not show a statistically significant relationship between the percentage of Ck-7 expression in tumor cells with primary, secondary, and total Gleason scores and Gleason grade groups of PAC. In other studies, it has been shown that Ck-7 expression is similar among adenocarcinomas with different Gleason scores (20, 24, 25). Only Goldstein *et al.* reported that Ck-7 immunohistochemical expression increased in higher Gleason scores. However, in their study, the increase in the expression of Ck-7 was in the range of rare expression to a maximum of less than 26% of tumor cells (19). Therefore, almost all researchers agree that a substantial increase in Ck-7 expression is not characteristic of PAC with higher Gleason scores. In general, the level of expression of Ck-7 in PAC tumor cells is insignificant and independent of the degree of tumor differentiation. Anyway, the difference in the staining instructions, the way of interpreting the results, and individual diversity among populations cause controversies in the results of published studies.

Based on the findings of our study, the expression of Ck-19 was observed in about 82% of the examined PAC samples. The average expression of Ck-19 in all tumor cells was about 41% and in the range of 0 to 78%. Other studies in this field have also reported the high frequency of Ck-19 expression among PAC patients. In Winter's study in Germany, which examined lymph nodes involved in PAC with a Gleason score higher than 7, Ck-19 expression was present in 95% of the samples (26). In another study conducted by Menz* et al.* in Germany, the expression of Ck-19 varied between 82.1% in tumor cells with a Gleason score of 10 to 92.8% in tumor cells with a Gleason score of 6 (27). However, contrary to the results of this study, the expression level of Ck-19 was significantly higher in tumor cells with higher primary, secondary, and total Gleason scores and Gleason grade groups. So, the average expression percentage of Ck-19 increased from 26.25% to 66.5% of tumor cells with an increasing Gleason score of 4 to 9. Also, the lowest level of expression of this marker was 17.3% in tumor cells, with a total Gleason score of 5 observed. All samples negative for Ck-19 also had 1 Gleason grade group or total Gleason scores of 4 to 6.

Previous studies have reported that as epithelial tumors increase in size, tumor prognosis worsens and is usually accompanied by the increased loss of differentiation, which is associated with increased expression of Ck-19 (28). The association of Ck-19 expression with tumor size, progression, and poor prognosis in breast cancer has also been reported (29). However, in the study of Winter* et al.*, the expression level of Ck-19 in PAC was not related to the Gleason score (26). Ck-19 expression levels are associated with several key tumor molecular features such as estrogen and progesterone receptor expression in breast cancer, and von Hippel-Lindau gene alterations in kidney tumors, suggesting that altered cellular functions or the differentiation status of neoplastic cells can be as easily influenced by expression levels and filament composition such as Ck-19 (27). The results of a study showed that the expression of Ck-19 in normal adult prostate glands is limited to the basal part, but in the fetal prostate, which has a lower degree of differentiation, the staining pattern of this marker is different and all cells, even luminal cells, are strongly positive for Ck-19 (30). Therefore, according to the results of our study, with the reduction of glandular differentiation in PAC, the expression of Ck-19 in these cells increases; however, the results of Menz* et al.*'s study do not confirm this finding. Of course, in the aforementioned study, in addition to the percentage of stained cells, the intensity of staining also played a role in defining the positivity of Ck-19 (27). In general, the use of different guidelines, antibodies, and interpretation criteria, as well as the difference in the thresholds used to define the positive expression of Ck-19, can cause differences in the results of published studies. Therefore, it is expected that different laboratory conditions can affect the rate of Ck-19 positivity. However, due to the limited information in this field, it is necessary to conduct more studies for definitive conclusions.

The findings of the present study did not show a statistically significant relationship between the age of patients with PAC and the expression percentage of Ck-7 and Ck-19 markers in the tumor cells. Therefore, it seems that the immunohistochemical expression of Ck-7 and Ck-19 markers in PAC is independent of the patient’s age and may be influenced by other factors. Anyway, considering that the relationship between age and the expression of these markers in PAC has not been investigated in previous studies, a definitive conclusion in this field requires more research.

**Limitations**

The main limitation of the current study is its retrospective nature, which causes the failure to examine the relapse and mortality status of patients and its relationship with the expression status of Ck-7 and Ck-19 markers to determine the effective role of these markers on prognosis. Therefore, conducting prospective studies in this field can be valuable in confirming or rejecting these results. Also, due to the presence of false positives and negatives during immunohistochemical staining, it is helpful to investigate the expression status of Ck-19 with more accurate methods and at the level of gene expression for more precise conclusions.

## Conclusion

Based on the findings of the present study, it can be concluded that PAC usually lacks the expression of Ck-7, and this marker is rarely, weakly, and focally expressed in these tumors, and its expression is independent of the Gleason score and Gleason grade group. In contrast, the expression of Ck-19 is increased in PAC, and most samples show strong expression of this marker. Also, with the increase of Gleason score and Gleason grade group and the loss of glandular differentiation in the tumor, the expression of Ck-19 increases, and in tumors with higher Gleason scores and Gleason grade groups, there is usually a strong expression of this marker. However, determining the relationship between the expression of this marker and the long-term survival of patients requires more studies.

## Ethical Approval

The study protocol was approved by the Ethics Committee of Shahed University (IR.SHAHED.REC.1400.122).

## Authors’ Contribution

Masood Soltanipur and Mohammadreza Jalali Nadoushan extracted data and samples were reviewed by Mohammadreza Jalali Nadoushan Also, statistical analysis was done by Hossein Yarmohammadi. The initial draft was written by Masood Soltanipur and Hossein Yarmohammadi which was finalized by Mohammadreza Jalali Nadoushan.

## Conflict of Interests

The authors confirm that there are no known conflicts of interest associated with this publication, and there has been no significant financial support for this work that could have influenced its outcome.

## References

[1] Siegel RL, Miller KD, Fedewa SA, Ahnen DJ, Meester RG, Barzi A (2017). Colorectal cancer statistics, 2017. CA: Cancer J Clin..

[2] Gandaglia G, Leni R, Bray F, Fleshner N, Freedland SJ, Kibel A (2021). Epidemiology and prevention of prostate cancer. Euro Urol Oncol.

[3] Tomlins SA, Rubin MA, Chinnaiyan AM (2006). Integrative biology of prostate cancer progression. Annu Rev Pathol Mech Dis..

[4] Murray TB (2021). The pathogenesis of prostate cancer. Exon Publications.

[5] Sehn JK (2018). Prostate cancer pathology: recent updates and controversies. Missouri Med..

[6] Aghamir SMK, Shafiee G, Ebrahimi M, Yarmohammadi H, Razmande R, Ahmadi H (2019). Comparison on diagnostic accuracy of prostate cancer detection tools: a systematic review and meta-analysis. Transl Res Urol..

[7] Dere Y, Altınboğa AA, Yaldır E, Bal K, Tosun K, Sarı A (2018). Correlation of Prognostic Gleason Grade Grouping and Histopathological Parameters: Can the" New System" Reflect the Pathological Perspective for Prognosis?.

[8] Lattouf JB, Saad F (2002). Gleason score on biopsy: is it reliable for predicting the final grade on pathology?. BJU Int..

[9] Hammerich KH, Ayala GE, Wheeler TM (2008). Application of immunohistochemistry to the genitourinary system (prostate, urinary bladder, testis, and kidney). Arch Pathol Lab Med..

[10] Eggener SE, Rumble RB, Armstrong AJ, Morgan TM, Crispino T, Cornford P (2020). Molecular biomarkers in localized prostate cancer: ASCO guideline. J Clin Oncol..

[11] Chu P, Wu E, Weiss LM (2000). Cytokeratin 7 and cytokeratin 20 expression in epithelial neoplasms: a survey of 435 cases. Mod Pathol..

[12] Cooper D, Schermer A, Sun TT (1985). Classification of human epithelia and their neoplasms using monoclonal antibodies to keratins: strategies, applications, and limitations. Lab Invest..

[13] Dum D, Menz A, Völkel C, De Wispelaere N, Hinsch A, Gorbokon N (2022). Cytokeratin 7 and cytokeratin 20 expression in cancer: A tissue microarray study on 15,424 cancers. Exp Mol Pathol..

[14] Lam K, Lui M, Lo C (2001). Cytokeratin expression profiles in thyroid carcinomas. Euro J Surg Oncol..

[15] Xin L (2019). Cells of origin for prostate cancer. Prostate Cancer..

[16] Gheitasi R, Sadeghi E, Jafari M (2021). Comparison of immunohistochemistry expression of CK7, HMWK and PSA in high-grade prostatic adenocarcinoma and bladder transitional cell carcinoma. Iran J Pathol..

[17] Mhawech P, Uchida T, Pelte M-F (2002). Immunohistochemical profile of high-grade urothelial bladder carcinoma and prostate adenocarcinoma. Hum Pathol..

[18] Adisa JO, Egbujo EC, Ibrahim B, Musa B, Madukwe J (2015). Expression of some selected cytokeratins and Ki67 protein in prostatic tumor: can these be used as tumor markers. Pan Afr Med J..

[19] Goldstein NS (2002). Immunophenotypic characterization of 225 prostate adenocarcinomas with intermediate or high Gleason scores. Am J Clin Pathol..

[20] Genega E, Hutchinson B, Reuter V, Gaudin P (1999). Immunophenotype of intermediate and high grade prostatic and urothelial carcinoma. Mod Pathol..

[21] Bassily NH, Vallorosi CJ, Akdas G, Montie JE, Rubin MA (2000). Coordinate expression of cytokeratins 7 and 20 in prostate adenocarcinoma and bladder urothelial carcinoma. Am J Clin Pathol..

[22] Kunju LP, Mehra R, Snyder M, Shah RB (2006). Prostate-specific antigen, high-molecular-weight cytokeratin (clone 34βE12), and/or p63: an optimal immunohistochemical panel to distinguish poorly differentiated prostate adenocarcinoma from urothelial carcinoma. Am J Clin Pathol..

[23] Dariane C, Clairefond S, Péant B, Communal L, Thian Z, Ouellet V (2022). High Keratin-7 Expression in Benign Peri-Tumoral Prostatic Glands Is Predictive of Bone Metastasis Onset and Prostate Cancer-Specific Mortality. Cancers..

[24] Cheville JC, Dundore PA, Bostwick DG, Lieber MM, Batts KP, Sebo TJ (1998). Transitional cell carcinoma of the prostate: clinicopathologic study of 50 cases. Cancer..

[25] Nagle RB, Brawer MK, Kittelson J, Clark V (1991). Phenotypic relationships of prostatic intraepithelial neoplasia to invasive prostatic carcinoma. Am Pathol..

[26] Winter A, Engels S, Goos P, Süykers M-C, Henke R-P, Gerullis H (2018). Detection of CK19 mRNA using one-step nucleic acid amplification (OSNA) in prostate Cancer: preliminary results. J Cancer..

[27] Menz A, Bauer R, Kluth M, von Bargen CM, Gorbokon N, Viehweger F (2021). Diagnostic and prognostic impact of cytokeratin 19 expression analysis in human tumors: A tissue microarray study of 13,172 tumors. Hum Pathol..

[28] Mujyambere B, Jayaraj R, Suja S (2018). Cytokeratin 19 (CK19) as a marker for Epithelial Differentiation and Malignant Transformation: Its Clinical relevance in Diagnosis, Prognosis and Treatment response monitoring. Iconic Res Eng J..

[29] Brotherick I, Robson C, Browell D, Shenfine J, White M, Cunliffe W (1998). Cytokeratin expression in breast cancer: phenotypic changes associated with disease progression. Cytometry..

[30] Letellier G, Perez M-J, Yacoub M, Levillain P, Cussenot O, Fromont G (2007). Epithelial phenotypes in the developing human prostate. J Histochem Cytochem..

